# Multidisciplinary protocols are an important part of enhanced recovery after major lower extremity amputation

**DOI:** 10.3389/fsurg.2025.1637121

**Published:** 2025-10-02

**Authors:** Christian Campat, Leigh Ann O’Banion

**Affiliations:** Department of Surgery, University of California San Francisco-Fresno Branch Campus, Fresno, CA, United States

**Keywords:** vascular surgery, CLTI, major lower extremity amputation, ERAS (enhanced recovery after surgery), multidiscipliary team

## Abstract

Major lower extremity amputation (MLEA) remains a high-risk procedure with significant implications for patient morbidity, mortality, and long-term functional independence. Optimizing outcomes for this vulnerable population requires a comprehensive, multidisciplinary approach guided by evidence-based perioperative care pathways. Enhanced Recovery After Surgery (ERAS) protocols offer a structured framework to improve recovery trajectories by standardizing key elements of care. This review examines the application of ERAS principles to the MLEA population, synthesizing current literature on preoperative assessment and patient selection, multimodal analgesia, early mobilization strategies, and coordinated post-discharge planning. By integrating findings from consensus statements and pilot studies, we demonstrate how tailored ERAS-based multidisciplinary protocols can reduce complications, enhance functional recovery, and promote equity in outcomes. The development and implementation of such structured care pathways represent a critical step toward improving the standard of care for patients undergoing major lower extremity amputation.

## Introduction

Chronic limb-threatening ischemia (CLTI) represents the most severe manifestation of peripheral artery disease and affects nearly two million adults in the United States (US). Despite advancements in medical therapy and revascularization strategies, long-term outcomes for patients with CLTI remain poor, with five-year mortality rates ranging from 40% to 80% (1). Major lower extremity amputation (MLEA) remains a common and often unavoidable outcome, accounting for over half of all amputations performed in the US (2). The burden of disease is progressive: nearly one-third of patients with CLTI will develop contralateral limb involvement within two years, and up to half of those who undergo MLEA will require a contralateral amputation within three years (3). Projections estimate that more than 3.6 million Americans will be living with limb loss by 2050 (4, 5).

The consequences of MLEA extend well beyond the hospital. Only 15%–30% of patients achieve functional independence following amputation, and outcomes are particularly poor among vascular patients—who are often older, medically complex, socioeconomically disadvantaged, and have reduced baseline functional status (6–8). Racial and ethnic disparities further exacerbate this challenge; patients from minority backgrounds are two to four times more likely to undergo MLEA, underscoring the need for equitable, structured care pathways (9, 10).

**Figure 1:**
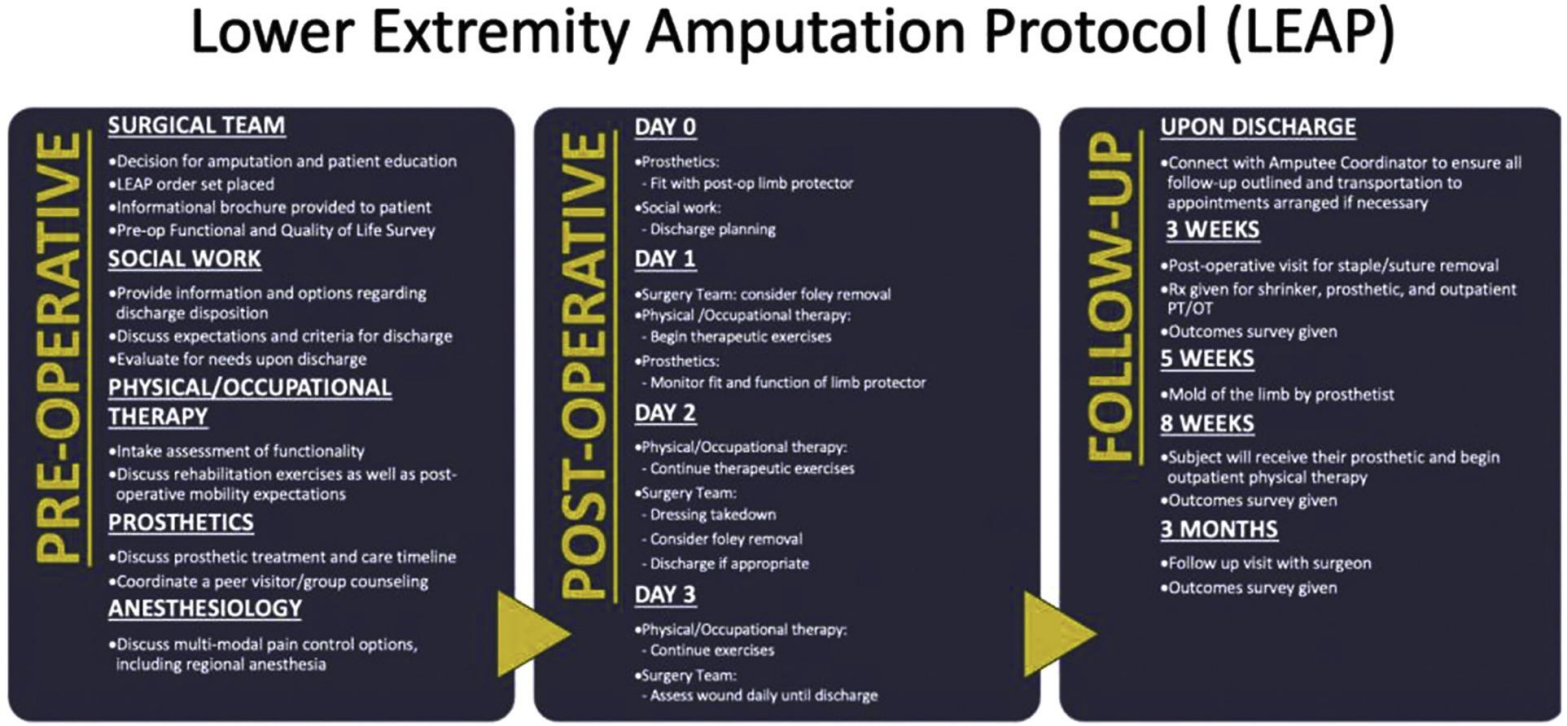
Implementation of ERAS principles into a multidisciplinary perioperative protocol for lower extremity amputation. Reproduced with permission of original authors. The Lower Extremity Amputation Protocol (LEAP): A Pathway to Successful Ambulation, O'Banion, Leigh Ann et al. Journal of Vascular Surgery, Volume 74, Issue 3, e27.

Optimizing outcomes after MLEA requires more than technical proficiency. It demands a multidisciplinary approach that supports patients across the continuum of care—from preoperative evaluation to postoperative rehabilitation. Surgeons, anesthesiologists, nursing staff, physical and occupational therapists, dietitians, prosthetists, and social workers must collaborate within standardized frameworks to ensure consistent, high-quality care (Figure 1). Enhanced Recovery After Surgery (ERAS) protocols offer such a framework, promoting early recovery, reducing complications, and improving functional outcomes through evidence-based perioperative strategies (11, 12). Core components—such as preoperative optimization, multimodal analgesia, early mobilization, and coordinated discharge planning—are particularly relevant for the MLEA population.

This review explores how ERAS principles can be applied to the care of patients undergoing major lower extremity amputation. By examining the available literature and established care models, we highlight the critical role of structured, multidisciplinary ERAS-based protocols in improving outcomes for this vulnerable and high-risk population.

## Pre-operative assessment and planning

Achieving a successful outcome following MLEA begins with diligent preoperative planning. This process must carefully balance the objectives of preserving functional limb length, promoting primary wound healing, and optimizing the potential for prosthetic rehabilitation. The ERAS framework provides a structured approach to this critical phase, emphasizing comprehensive risk stratification, functional evaluation, vascular assessment, and shared decision-making (12).

A thorough preoperative assessment starts with evaluation of systemic and psychosocial risk factors. Cardiovascular risk stratification is essential, given the high prevalence of coronary artery disease and congestive heart failure among patients with CLTI (13). Frailty screening should be performed as a routine part of preoperative workup, as diminished physiologic reserve has been independently associated with increased postoperative morbidity and mortality (14, 15). In addition, psychosocial risk factors—including preexisting mental health conditions, cognitive impairment, and risk of delirium—must be identified early to anticipate barriers to recovery (16). Social determinants of health also play a critical role in outcomes; patients with limited family or caregiver support may face challenges with post-discharge recovery, prosthetic use, and long-term rehabilitation. Engaging social work or case management services during the preoperative phase is crucial to anticipating these needs (17).

Assessment of functional status is equally important in amputation planning. Preoperative mobility, including the ability to transfer and ambulate with assistive devices, helps guide postoperative goals. The Medicare Functional Classification Level (K level) is a validated tool for estimating ambulation potential, scoring patients on a scale graded K0–K4. Patients classified as K0 are unlikely to benefit from limb preservation strategies while those of K4 are either able or have the potential for high impact ambulation with a prosthesis (18). Motivation and individual rehabilitation goals should be explicitly explored, as they influence both perioperative engagement and long-term adherence to therapy. Of particular importance is the status of the contralateral limb, as disease progression on the opposite side is common and can significantly impact overall mobility and independence.

Patients with a goal of ambulation should receive early engagement with a prosthetist pre-operatively to educate the patient regarding the expected peri-operative course and provide a limb protector to prevent trauma to the limb in the immediate post-operative phase. Inclusion of prosthetists a peri-operative protocol for MLEA has been associated with faster time to ambulation with a prosthetic (4).

Peer counselling and support should also be offered at this time as it has been shown to have many perceived benefits including social support, feeling of connectedness and knowledge sharing while also being cost effective (19, 20). Some prosthetists may offer peer support groups and counselling, further reducing cost. Psychological counselling with education with cognitive behavioral therapy and education on biofeedback have also been shown to improve outcomes (21).

The physical examination should focus on identifying local factors that affect surgical planning. The presence of active infection or sepsis may necessitate a staged approach beginning with a guillotine amputation for source control (22). Vascular assessment, both clinical and imaging-based, is critical to determining the optimal level of amputation. Patency of the profunda femoris artery and the popliteal artery are strong predictors of successful healing in trans-tibial amputation (TTA) (23). When available, CT angiography or duplex ultrasound can provide detailed information about inflow and runoff vessels, allowing for more precise surgical planning. Additional laboratory testing, including renal function, albumin, and hemoglobin A1c levels, offers further insight into the patient's capacity for healing.

Whenever feasible, TTA is preferred over trans-femoral amputation (TFA) due to improved postoperative mobility, reduced energy expenditure during ambulation, and greater potential for prosthetic rehabilitation (24). However, this preference must be tempered by realistic assessments of tissue viability, perfusion, comorbid conditions, and overall functional capacity. In some cases, an TFA may offer a more reliable pathway to recovery, particularly in non-ambulatory or frail patients or those with inadequate distal perfusion who are not candidates for revascularization.

Ultimately, the decision regarding level of amputation should be grounded in a shared decision-making process. Patients must be educated about their condition, treatment options, and expected outcomes, and they should be engaged as active participants in determining their care plan. Preoperative counseling, ideally involving family members or caregivers, reinforces understanding and fosters alignment of expectations. Empowering patients through education and shared decision-making not only enhances satisfaction but is also associated with improved adherence to postoperative rehabilitation and better long-term outcomes (12, 23).

## Inpatient perioperative care

The perioperative phase of care is critical in determining the trajectory of recovery for patients undergoing MLEA. Integration of ERAS principles into this phase has been shown to significantly improve patient outcomes by standardizing perioperative management and accelerating functional recovery. A recent consensus statement by the ERAS Society and the Society for Vascular Surgery (SVS) emphasized the importance of several key domains during this phase, including multimodal analgesia, evidence-based surgical techniques, early mobilization, mental health support, and coordinated rehabilitation planning (12). The successful implementation of these principles within a multidisciplinary protocol can optimize pain control, reduce complications, shorten hospital stays, and improve the likelihood of independent ambulation (25).

Effective analgesia is foundational to postoperative recovery and must begin in the preoperative period. Multimodal analgesic strategies are recommended, incorporating non-opioid agents such as non-steroidal anti-inflammatory drugs (NSAIDs), acetaminophen, and gabapentinoids, as well as regional anesthetic techniques including peripheral nerve blocks. Preoperative use of regional anesthesia not only improves immediate postoperative pain control but may also reduce opioid consumption and associated complications, particularly in older, frail patients with limited cardiopulmonary reserve (26–28). Postoperatively, a multimodal regimen should be continued to allow for early mobilization and participation in physical therapy. One of the most challenging aspects of pain management in this population is phantom limb pain (PLP), which affects a substantial number of amputees and can significantly impair rehabilitation (25, 26). While various pharmacologic agents—such as anticonvulsants, antidepressants, and opioids—have demonstrated some efficacy, their routine use remains controversial. Importantly, multidisciplinary strategies that incorporate anesthesiologists, physical therapists, and prosthetists into a coordinated perioperative plan have been associated with reduced incidence and severity of PLP (25). For patients with chronic pain post-amputation, individualized management strategies should be employed, which may include ongoing physical therapy to build strength and reduce prosthesis-related discomfort, as well as cognitive behavioral therapy or biofeedback techniques to address the psychological burden of limb loss. In refractory cases, targeted muscle reinnervation (TMR) may be considered (29, 30).

Early mobilization is another core component of ERAS protocols and is particularly vital for patients undergoing MLEA. Ideally, physical and occupational therapy (PT/OT) should begin prior to surgery when amputation is anticipated. Preoperative engagement with PT/OT provides an opportunity to assess baseline strength, educate patients about the recovery process, and begin preparing them for ambulation with a prosthesis (25). Following surgery, early mobilization should be prioritized to mitigate the adverse effects of prolonged bedrest such as muscle atrophy, venous thromboembolism, and deconditioning. Studies have shown that patients who participate in early physical therapy as part of a multidisciplinary ERAS-based protocol spend fewer days immobilized and are discharged more quickly from the hospital, with improved functional outcomes (6). A prosthetist should also be assessing the fit of the limb protector and adjust as needed to prevent trauma in this critical period of healing.

Discharge planning is a critical element of perioperative care and must be initiated early in the hospital course. A coordinated, multidisciplinary team—including case managers, social workers, and rehabilitation specialists—should assess each patient's post-discharge needs and identify the most appropriate discharge destination. For patients returning home, this may involve a home safety assessment, procurement of durable medical equipment, and coordination of home health services. However, most patients benefit from discharge to an acute inpatient rehabilitation hospital, where they can receive intensive PT/OT—up to six hours per day—and 24-hour medical supervision. While skilled nursing facilities (SNFs) may serve as appropriate discharge destinations for some individuals based on specific social or medical factors, outcomes data have demonstrated that patients discharged to SNFs tend to have higher 30-day readmission rates, longer time to functional recovery, and lower rates of independent ambulation compared to those discharged to acute rehabilitation hospitals (4, 25, 31). As such, every effort should be made to facilitate access to high-intensity rehabilitation environments for patients with the potential to ambulate post-MLEA.

Together, these perioperative strategies form the cornerstone of successful recovery following MLEA. When implemented through a multidisciplinary, ERAS-based framework, they not only improve clinical outcomes but also support patient autonomy, promote earlier return to independence, and reduce overall healthcare burden.

## Post-discharge care and longitudinal follow-up

Post-discharge care is a critical phase in the recovery process for patients undergoing MLEA, with significant implications for wound healing, prosthetic fitting, functional mobility, and long-term independence. A well-defined and proactive follow-up plan ensures that patients meet essential clinical and rehabilitative milestones while also providing opportunities for early identification and management of complications.

Standardized, multidisciplinary limb amputation protocols that incorporate routine follow-up have been shown to significantly reduce the time to prosthesis acquisition and improve functional outcomes (4). Early outpatient follow-up with the operating surgeon typically occurs within three weeks of discharge and focuses on residual limb/wound evaluation and pain assessment. This visit also provides an opportunity to reassess vascular status of both limbs and ensure the absence of complications (infection, dehiscence, or ischemia). A second surgical follow-up at approximately three months allows for evaluation of the residual limb and contralateral leg, ensuring that patients are appropriately progressing toward rehabilitation goals.

Ongoing collaboration with a prosthetist is essential during this phase. Prosthetists assess the fit and function of the prosthesis, make necessary adjustments, and monitor for limb volume changes that can impact socket fit and skin integrity (18). Education on prosthetic hygiene, skin care, and pressure injury prevention is also a vital component of prosthetic follow-up. The prosthetist may provide a limb shrinker after the surgical wound is healed, usually around three weeks post op. Molding for a long-term prosthesis and gait training with the prosthetist can occur as soon as five weeks post op with delivery of the final prosthetic ideally occurring around week eight. Open and regular communication between the surgical and prosthetic teams is critical to rapidly identifying and addressing barriers to optimal device use and ensuring continuity of care.

Physical therapy must also continue during the post-discharge period to support gait training, balance, endurance, and strength-building. A standardized protocol that integrates surgical, prosthetic, and rehabilitation follow-up not only strengthens continuity of care but also fosters trusting relationships between patients and providers (4).

This comprehensive, team-based approach to post-discharge care reinforces the core principles of ERAS and ensures that patients remain supported as they transition from acute recovery to long-term independence. By maintaining structured and interdisciplinary follow-up, healthcare teams can improve prosthesis utilization, reduce complications, and optimize quality of life for patients recovering from MLEA.

## Challenges in implementation

While the adoption of ERAS protocols for MLEA offers significant potential to improve outcomes, the implementation of these multidisciplinary care models is not without challenges. Healthcare institutions may be hesitant to commit the necessary resources—such as dedicated personnel, equipment, and infrastructure—required to establish and sustain such programs. This reluctance may stem from competing institutional priorities or concerns about upfront costs. However, growing evidence supports the cost-effectiveness of ERAS protocols in this context, as they are associated with reduced length of hospital stay, lower complication rates, and improved rehabilitation trajectories, all of which contribute to downstream cost savings and more efficient use of healthcare resources (32).

Beyond institutional considerations, the successful implementation of ERAS-based protocols must also address the broader social determinants of health that influence patient outcomes. Many patients undergoing MLEA face socioeconomic barriers, limited health literacy, and disparities in access to high-quality perioperative care. These factors can impact everything from preoperative optimization to post-discharge rehabilitation. To address these inequities, educational materials should be culturally sensitive and available in multiple languages. Incorporating community health workers or patient advocates into the care team may help bridge communication gaps and ensure patient engagement across diverse populations.

Successful implementation depends on strong leadership, interprofessional collaboration, and sustained investment in quality improvement. Pilot programs that demonstrate early wins—such as reduced readmissions or shorter time to ambulation—can help build institutional support and serve as models for broader adoption (25). Ultimately, the widespread success of ERAS protocols in the MLEA population will require not only clinical rigor but also an intentional effort to build systems that are equitable, inclusive, and responsive to the needs of vulnerable patients.

## Conclusion

Implementing an ERAS-based protocol for patients undergoing major lower extremity amputation is essential to improving functional outcomes and quality of life. These multidisciplinary pathways support coordinated care across the perioperative continuum—beginning with preoperative education and patient-centered decision-making, continuing through pain management and early mobilization, and extending into discharge planning and long-term follow-up. When tailored to the individual's goals and supported by a committed team, ERAS protocols empower patients to achieve optimal recovery and greater independence after amputation.
